# Comparative Study Between Laparoscopic Transabdominal Preperitoneal Repair Plus and Laparoscopic Intraperitoneal Onlay Repair Plus of Umbilical and Paraumbilical Hernia

**DOI:** 10.3389/jaws.2025.14663

**Published:** 2025-06-03

**Authors:** Karthik Munjuluri, Abirami J. Raghunath, Naveen Alexander

**Affiliations:** Department of General Surgery, Sri Ramachandra Institute of Higher Education and Research, Chennai, India

**Keywords:** laparoscopic, umbilical hernia, TAPP+, IPOM+, paraumbilical hernia

## Abstract

Laparoscopic ventral hernia repair has evolved to minimize the morbidity and recurrence rates associated with traditional open repairs. As laparoscopic expertise grows and newer mesh materials are developed, these techniques have become increasingly accepted due to the advantages of minimally invasive surgery. In laparoscopic hernia repair, mesh placement can either be intraperitoneal or preperitoneal. Intraperitoneal Onlay Mesh (IPOM+) placement brings the mesh into direct contact withabdominal contents, potentially leading to complications such as chronic pain, intestinal obstruction, fistula formation, infertility, and adhesions. To counteract these issues, composite meshes combining polypropylene with inert substances like collagen or cellulose have been introduced, though their high cost remains a challenge. An alternative approach, Transabdominal Preperitoneal (TAPP+) repair, uses a less expensive polypropylene mesh placed in the preperitoneal space, minimizing adhesion formation and mesh-related complications. However, the TAPP+ procedure is technically more demanding and can result in longer operative times. This study compares the safety and efficacy of TAPP+ and IPOM+ techniques in repairing umbilical and paraumbilical hernias, with emphasis on economical aspects.

## Introduction

The adoption of laparoscopic techniques in ventral hernia repair aims to mitigate the heightened morbidity and recurrence rates associated with traditional open repair methods. Over time, as laparoscopic expertise has grown and newer meshes have been developed, this approach has gained acceptance and has the potential to become the preferred procedure because of the benefits of minimally invasive surgery [1–3]. During laparoscopic repair, the mesh is positioned either intraperitoneally or in the peritoneal/retromuscular spaces. The uniform distribution of intra-abdominal pressure across each square inch of the mesh, rather than along suture lines, as in conventional repair, contributes to the strength of the repair and reduces recurrence rates [1].

In intraperitoneal onlay mesh (IPOM+) placement, direct contact between the mesh and the abdominal contents cannot be avoided. Although a polypropylene mesh is cost-effective and integrates well into the abdominal wall, it triggers significant inflammatory reactions and adhesions. These complications, including chronic pain, intestinal obstruction, fistula formation, infertility, and surgical challenges, have led to the development of new-generation composite meshes [3–5]. These composite meshes combine conventional materials, such as polypropylene, with inert substances, such as collagen or cellulose, reducing bowel adhesions and the risk of fistulas. However, their high costs present challenges. As an alternative, transabdominal preperitoneal (TAPP+) repair using a less expensive polypropylene mesh was proposed [6, 7].

Preperitoneal retromuscular placement of a polypropylene mesh minimises adhesion formation and postoperative complications, leveraging the peritoneum as a protective barrier between the mesh and bowel. This approach ensures effective abdominal wall reinforcement due to immediate mesh fixation under intra-abdominal pressure while also avoiding intraperitoneal mesh-related complications and fixation device issues. Despite its advantages, the preperitoneal approach may require longer operative times for dissection and the development of the pre peritoneal plane for mesh placement [8].

Umbilical hernias are relatively common in Western countries and affect up to 2% of the adult population. Despite its prevalence, the most commonly performed technique for repair is the open anterior approach. This preference is largely due to the ability to make a small incision in the skin and the relatively short duration of the surgical procedure. However, this method may pose a higher risk of hernia recurrence, particularly because it often lacks reinforcement of defects with synthetic materials [1–4].

For small umbilical hernias measuring 1–2 cm, the risk of recurrence is three times higher when mesh implantation is not utilised. The likelihood of recurrence also rises with the size of the hernia orifice and patient’s body mass index. Given the increasing number of overweight and obese individuals in the population, the demand for umbilical hernia treatments is expected to rise. These patients, who are at a higher risk of diastasis recti (separation of the abdominal muscles), are also more prone to midline hernia recurrence [5].

Surgeons should consider reinforcing the hernia defect with synthetic mesh if recti divarication is confirmed. Additionally, overweight and obese patients face a higher risk of complications, particularly surgical site infections, which further increase when using an anterior approach with mesh. Laparoscopic hernia repair is recommended to mitigate the risk of infection and prevent recurrence.

This study aims to evaluate the efficiency, postoperative pain management, duration of recovery, and potential complications between Transabdominal Preperitoneal Repair Plus (TAPP+) and Laparoscopic Intraperitoneal Onlay Mesh Repair Plus (IPOM+) for umbilical and paraumbilical hernias, specifically comparing the duration of surgery, postoperative pain and requirement of analgesics, duration of hospital stay, seroma formation and cost of surgery.

## Methodology

### Study Design

This prospective observational study was conducted at the Department of General Surgery, Sri Ramachandra Institute of Higher Education and Research, Porur, Chennai. The study period spanned for a period of 3 years and involved patients with umbilical and paraumbilical hernias.

### Aim and Objectives

To compare the outcome of Transabdominal preperitoneal repair plus (TAPP+) and laparoscopic intraperitoneal onlay repair plus (IPOM +) of umbilical and paraumbilical hernia with regards to

1. Duration of Surgery
2. Postoperative pain on Day 0, 1, 7, 14 and 30
3. Duration of stay in hospital
4. Seroma formation
5. Cost of Surgery

### Study Population

#### Inclusion Criteria

The study included patients aged more than 18 years who presented with a defect size of 4 cm or less, with single primary umbilical or paraumbilical hernias, and were scheduled for elective surgery. Patients who met the inclusion criteria were enrolled after obtaining informed consent.

#### Exclusion Criteria

Exclusion criteria included defect sizes greater than 5 cm, multiple defects, recurrent hernias, muscular repairs, and cases requiring emergency intervention or concomitant procedures. Patients who were medically unfit for general anaesthesia were also excluded.

### Data Collection

The study involved 50 patients, of which 33 underwent intraperitoneal onlay meshplasty plus (IPOM+) and 27 underwent transabdominal preperitoneal repair (TAPP+).

Basic information such as age, sex, and hernia defect size was recorded for all volunteers. Detailed medical histories, clinical examinations, and relevant investigations were documented and recorded.

### Surgical Technique

Patients underwent either transabdominal preperitoneal repair plus (TAPP+) or Laparoscopic Intraperitoneal Onlay Repair Plus (IPOM+). The outcomes of these two techniques were compared based on several parameters.

### Outcome Measures

The primary outcomes were as follows.

1. Duration of Surgery: Measured from the start time to the end of surgery in minutes.
2. Postoperative Pain: Assessed on days 0, 1 and 7 using the Visual Analogue Scale (VAS). All patients received standard postoperative analgesia protocol, and additional requirement of analgesics was recorded. Post operative pain was further assessed using VAS on follow-up on day 14, and day 30, and on outpatient basis upto a duration of 3 months postoperatively.
3. Seroma Formation: This is defined as the development of a serous pocket of fluid, and was assessed clinically, in patients who presented with swelling at the site of surgery postoperatively. If present this was managed conservatively with compression dressings.
4. Duration of Hospital Stay: From the time of admission till discharge from the hospital.
5. Cost of Surgery: Includes the mesh and tackers used for the procedures.

### Follow-Up and Data Analysis

Patients were followed-up to monitor the duration of hospital stay and postoperative pain. Seroma formation was also clinically assessed and recorded. All collected data were analysed to establish the percentage of outcomes related to TAPP+ and laparoscopic intraperitoneal onlay repair. This analysis aimed to compare the efficacy and safety of the two surgical techniques.

Statistical analysis was performed using SPSS software, version 16.0. Continuous variables were tested for normality using the Shapiro–Wilk test. Variables following a normal (Gaussian) distribution were summarized as mean ± standard deviation (SD), while those not normally distributed were expressed as median with interquartile range (IQR). Categorical variables were presented as frequencies and percentages. Comparisons between the two groups were conducted using the independent samples t-test for normally distributed continuous variables, and the Mann–Whitney U test for non-normally distributed data. Categorical variables were compared using either the Chi-square test or Fisher’s exact test, depending on the expected cell counts. A two-tailed p-value of less than 0.05 was considered statistically significant.

### Ethical Considerations

This study was conducted in compliance with ethical standards. Informed consent was obtained from all participants and the study protocol was reviewed and approved by the institutional review board.

### Surgical Technique

**FIGURE 1:**
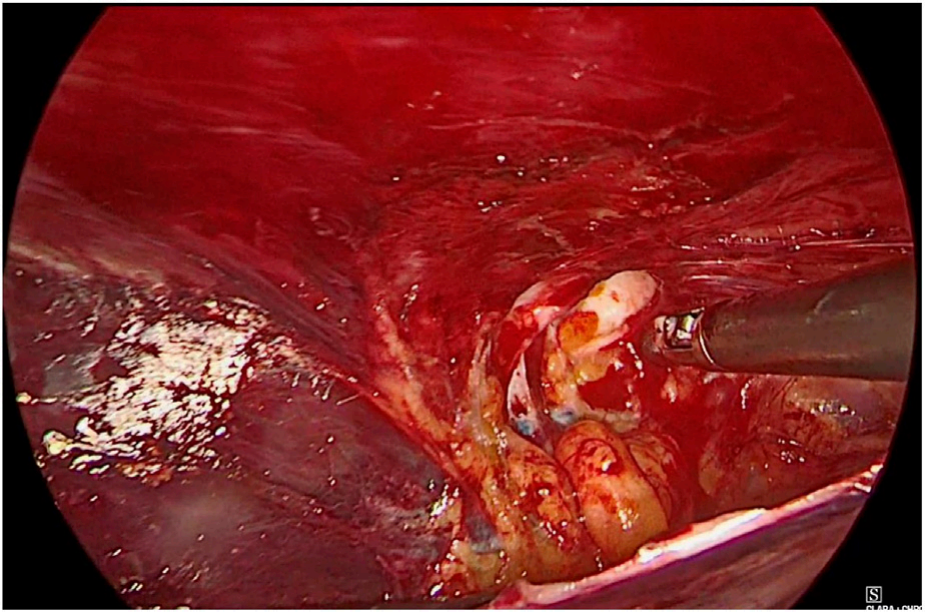
Hernial sac with content.

**FIGURE 2:**
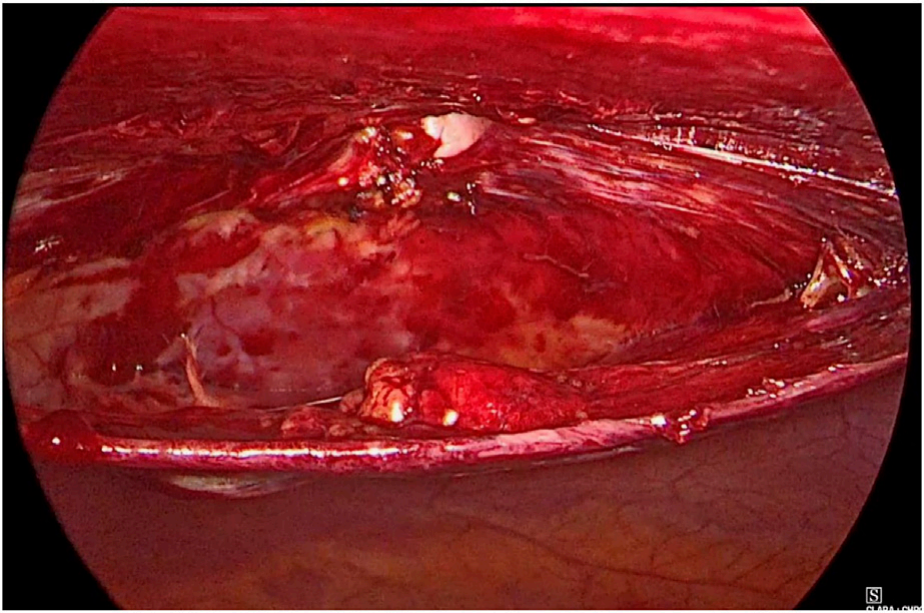
Hernial content reduced.

**FIGURE 3:**
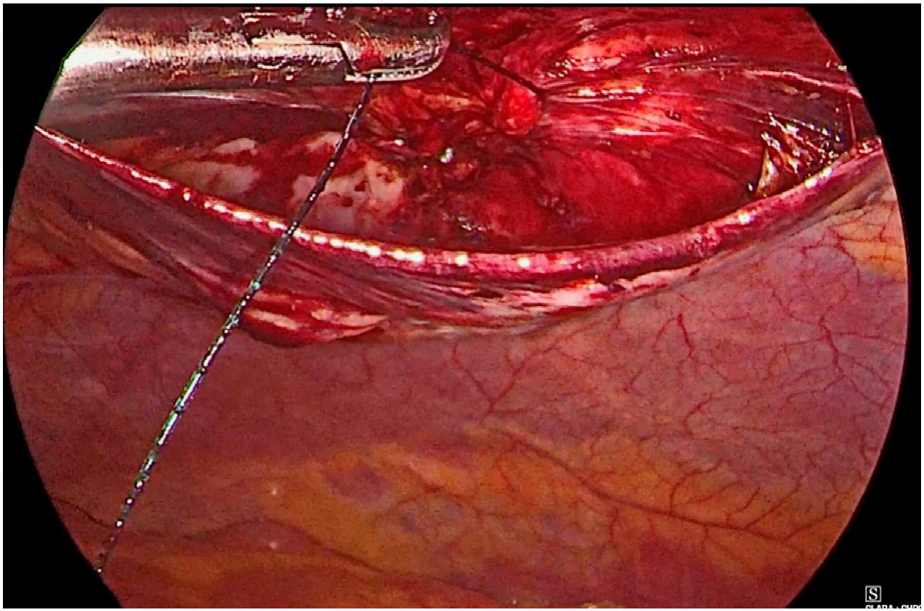
Defect closure using barbed suture.

**FIGURE 4:**
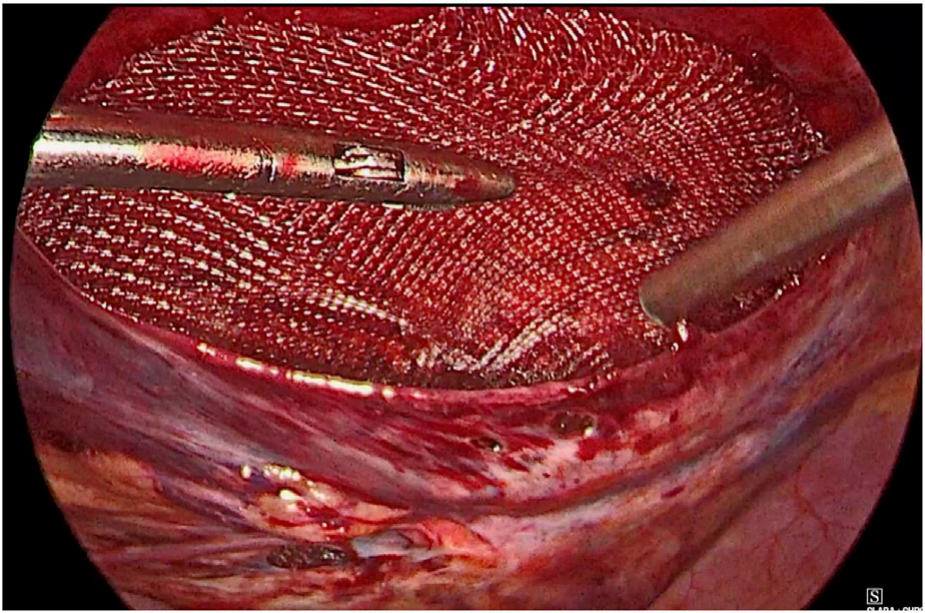
Mesh placed inside pre peritoneal space.

TAPP+: The procedure begins with access to the peritoneal cavity. After establishing a Pneumoperitoneum of 15 mmHg, an overview of the abdominal cavity was also obtained. Adhesions are released. The peritoneum is grasped at least 7 cm from the hernia defect and incised at the left paramedian line, this is done using monopolar scissors. The hernia sac with the herniated tissue is released and retracted into the intra-abdominal cavity. To facilitate the mesh placement over the defect, a pre-peritoneal area of at least 5 cm in all directions is raised and prepared. Primary closure of the hernia defect is performed using non- absorbable barbed sutures. For this step, intra-abdominal pressure is reduced to 8–10 mmHg. Next, the mesh is positioned between the posterior rectus sheath and peritoneum. Like the mesh placement in inguinal TAPP+ repair, no securing sutures to the mesh are necessary. And if the peritoneum is injured during the preparation, these are repaired with absorbable sutures. After the mesh is adequately positioned, the peritoneal flap is closed with an absorbable barbed suture or tackers. The trocars are removed under visual and the pneumoperitoneum is reduced (Figures 1–4).

**FIGURE 5:**
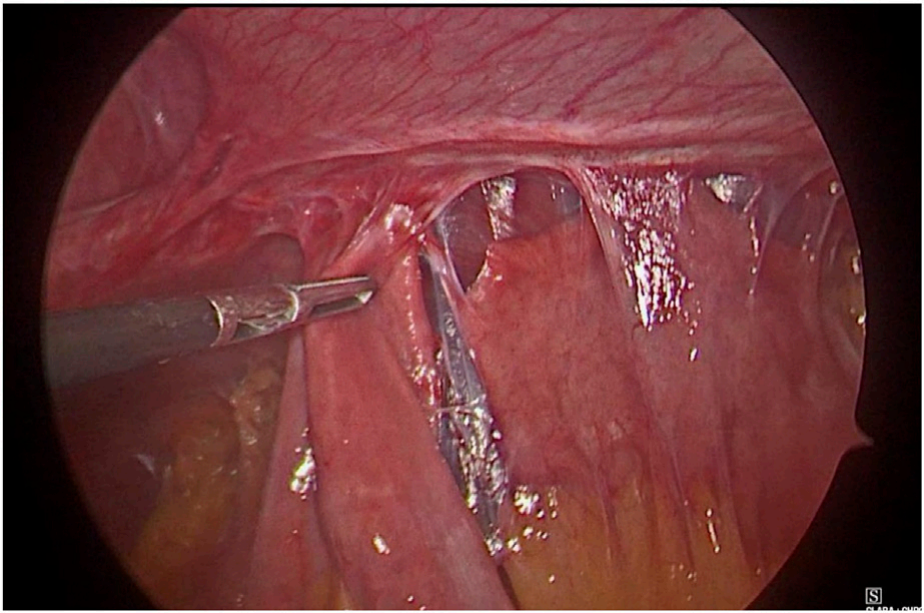
Adhesiolysis.

**FIGURE 6:**
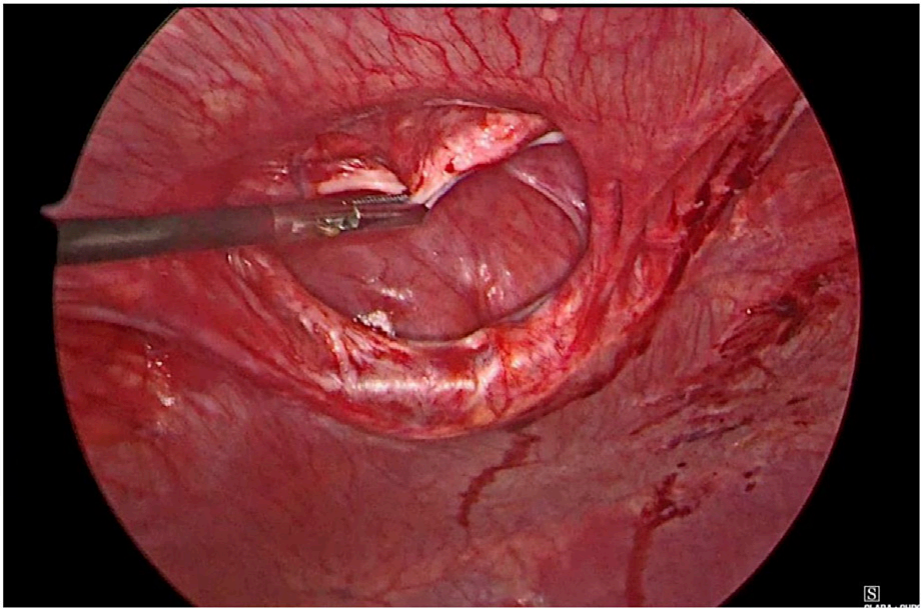
Hernial defect with sac.

**FIGURE 7:**
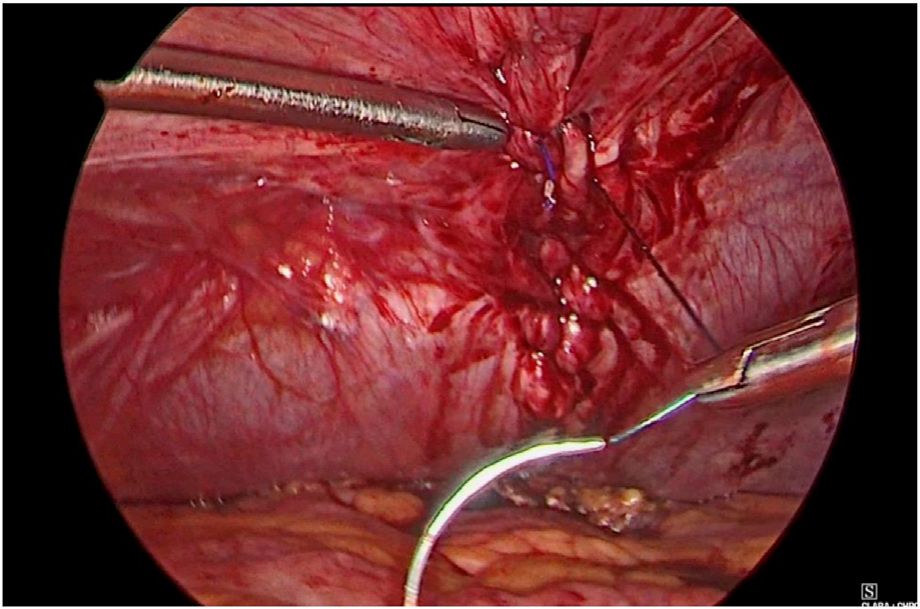
Defect closure using barbed sutures.

**FIGURE 8:**
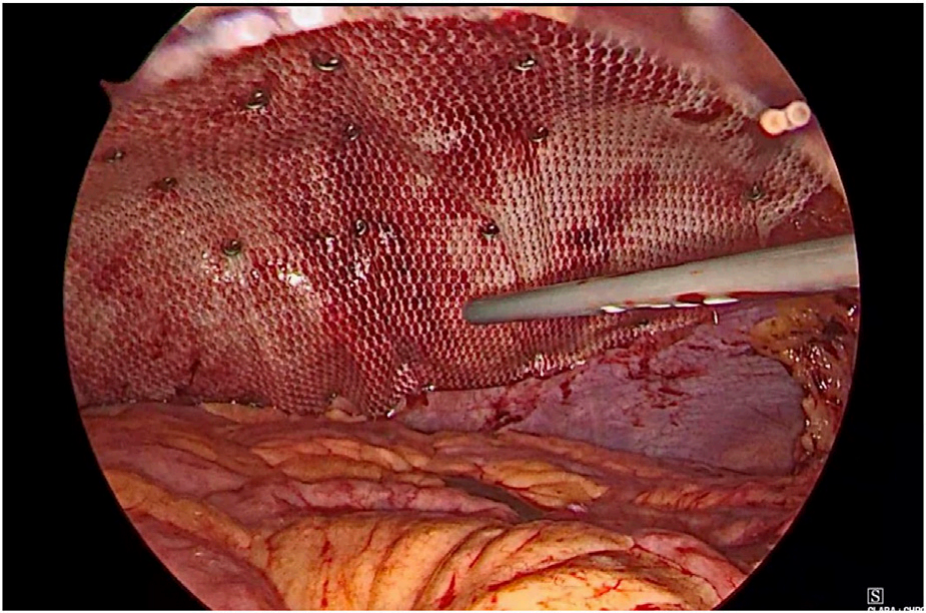
Composite mesh fixation using tackers.

IPOM+: The patient is positioned supine, and pneumoperitoneum is established. A 30° laparoscope is used to access the hernia defect. After 360° inspection of the abdominal cavity, all abdominal wall adhesions, if present, are released. After identifying, the hernia contents are reduced into the abdominal cavity. Structures surrounding the defect and possibly obstructing mesh placement, such as the peritoneum or the umbilical and falciform ligaments, are dissected. The fascial defect is measured under vision. The primary closure of the hernia defect is performed with non-absorbable barbed sutures, constitutng the “plus” technique. The mesh is then deployed and fixed intraperitoneally using absorbable staples in a “double crown” technique, with a minimum of 5 cm overlap to reduce recurrence risk (Figures 5–8).

## Results

The distribution of patients by age group showed no statistically significant difference between the IPOM+ and TAPP+ groups (p = 0.376). In the IPOM+ group, 6.1% were under 30 years, 24.2% were between 31 and 40 years, 39.4% were between 41 and 50 years, 18.2% were between 51 and 60 years, and 12.1% were over 61 years of age. In the TAPP+ group, 3.7% were under 30 years old, 40.7% were between 31 and 40 years, 22.2% were between 41 and 50 years, 11.1% were between 51 and 60 years, and 22.2% were over 61 years old.

There was a significant difference in the sex distribution between the IPOM+ and TAPP+ groups (p = 0.013). In the IPOM+ group, 75.8% of the patients were female and 24.2% were male. In the TAPP+ group, 44.4% were female and 55.6% were male.

The mean defect size was recorded taking the largest diameter of the defect into consideration, based on which the overlap of mesh required was also decided. The mean defect size was slightly larger in the IPOM+ group with largest diameter of 2.35 cm (SD = 0.92), as compared to the TAPP+ group with 2.06 cm (SD = 0.73), but this difference was not statistically significant on analysis (p = 0.18).

### Duration of Surgery

The duration of surgery was significantly longer in the TAPP+ group than that in the IPOM+ group. The mean duration was 79.26 min (SD = 8.55) for TAPP+ and 70.21 min (SD = 11.90) for IPOM+, with a p-value of 0.002 (Table 1).

**TABLE 1:** Comparison of various factors between TAPP+ and IPOM+ groups.

Factors assessed	TAPP+ Group	IPOM+ Group	P-value
Duration of surgery (minutes)	70.21	79.26	0.002
Pain score day 0 (mean VAS)	3.48	4.21	0.002
Pain score day 1 (mean VAS)	1.52	1.91	0.002
Seroma	0	6.1%	0.193
Cost of mesh (RS)	7,444.44	27,878.79	<0.0001
Cost of tackers (RS)	15,444.44	22,121.21	<0.0001

### Duration of Hospital Stay

The mean duration of postoperative hospital stay was 1.8 ± 0.6 days in the TAPP+ group and 2.3 ± 0.7 days in the IPOM+ group. The difference was not statistically significant (p = 0.12), suggesting comparable recovery profiles between the two approaches.

### Post Operative Pain Scores

Patients were assessed on postoperative days 0, 1, 7, 14 and 30 using the Visual Analogue Scale (VAS). All patients received standard postoperative analgesia protocol, with local anaesthesia infilatration at the port sites intraoperatively, and intravenous paracetamol injections three times a day until postoperative day 1. Any additional requirement of intravenous analgesics was recorded. Chronic pain was further assessed on outpatient basis upto a duration of 3 months postoperatively.

Patients in the IPOM+ group reported higher pain scores on postoperative day 0 than those in the TAPP+ group did. The mean pain scores were 4.21 (SD = 0.86) for the IPOM+ and 3.48 (SD = 0.89) for the TAPP+, with a statistically significant p-value of 0.002 (Table 1). On postoperative day 1, pain scores remained significantly higher in the IPOM+ group with a mean of 1.91 (SD = 0.58) compared to 1.52 (SD = 0.58) in the TAPP+ group, with a p-value of 0.012. On postoperative day 7, the pain scores were comparable between the two groups, and thereafter completely resolved by postoperative day 14. There were no cases of chronic pain or neuralgia in either group.

### Seroma Formation

Seroma was assessed clinically, in patients who presented with swelling at the site of surgery postoperatively. This was only managed conservatively with compression dressings, and on further follow up, completely resolved. Aspiration was not attempted in any patient developing a seroma. There were no hematomas and none of the patients developed surgical site infections. Seroma formation was not significantly different between the groups (p = 0.193). In the IPOM+ group, 6.1% of the patients developed seroma, whereas none of the patients in the TAPP+ group developed seroma (Table 1).

### Cost of Mesh and Tackers

The cost of mesh was significantly higher in the IPOM+ group with a mean of 27,878.79 INR (SD = 2011.80) than in the TAPP+ group, with a mean of 7,444.44 INR (SD = 4,660.25), with a p-value of <0.0001 (Table 1). Similarly, the cost of tackers was significantly higher in the IPOM+ group with a mean of 22,121.21 INR (SD = 2,858.73) compared to the TAPP+ group with a mean of 15,444.44 INR (SD = 9,254.24), with a p- value of <0.0001 (Table 1).

### Recurrence and Surgical Site Infections

At the 6-month follow-up, no cases of surgical site infection, seroma or hematoma were observed in either the IPOM+ or TAPP+ group. No short-term recurrences were noted.

## Discussion

Studies comparing TAPP+ and IPOM+ for umbilical and paraumbilical hernias have shown that the TAPP+ technique for umbilical hernia repair allows for the placement of a larger mesh than the anterior open approach, aligning more closely with current recommendations. This is particularly beneficial for patients with additional risk factors, such as obesity or diastasis recti. The TAPP+ method places the mesh in the preperitoneal space, avoiding direct contact with the bowel. Although the laparoscopic TAPP+ method is safe, it requires a longer operation time than open methods because of the dissection of the preperitoneal space and the hernial sac. Despite the longer procedure time, there were no significant differences in hospitalisation time, postoperative pain, or early recurrence between the TAPP+ and open ventral patch repair methods. Patients reported better cosmetic results with the ventral patch method but were highly satisfied with both treatments. Further analysis is required to determine the long- term effectiveness of these methods in preventing recurrence [9–11].

A prospective randomised trial by Sarli et.al, involving 115 patients with 148 hernias compared the TAPP+ and IPOM+ techniques for laparoscopic hernia repair. The study found that TAPP+ took significantly longer than IPOM+, but there were no intraoperative complications, conversions to open repair, or postoperative deaths in either group. Postoperative complications occurred in 16.9% of the TAPP+ patients and 25% of the IPOM+ patients, with neuralgia occurring more frequently in the IPOM+ group. Recurrences were reported in 11.1% of IPOM+ hernias but not in TAPP+ hernias. Another study highlighted the advantages of the TAPP+ technique in umbilical hernia repair, allowing for the placement of a larger mesh than anterior approach surgery, which aligns with current recommendations, especially for patients with additional risk factors like obesity or diastasis recti [12].

A comparative study of transabdominal preperitoneal versus intraperitoneal onlay mesh repair for laparoscopic ventral hernia repair found that both techniques are feasible and safe, but the TAPP+ method allows for the placement of a larger mesh and avoids direct contact between the mesh and intestines. This study suggested that the TAPP+ method might be more effective in preventing recurrence, especially in obese patients. These studies suggest that both the TAPP+ and IPOM+ techniques are safe and effective for umbilical and paraumbilical hernias, with TAPP+ offering advantages in terms of mesh size and placement, especially for patients with additional risk factors. However, TAPP+ has a longer operation time than IPOM+, and the choice of technique may depend on the surgeon’s experience and individual patient’s needs [13].

Our study reports that the analysis of TAPP+ and IPOM+ for umbilical and paraumbilical hernias demonstrated that TAPP+ is associated with lower postoperative pain and reduced costs for mesh and tackers, despite a longer operative time. A similar study finding was also reported by Megas et al., who reported that Ventral-TAPP+ procedures represent an alternative technique to laparoscopic IPOM+ repair to reduce the risk of complications associated with the intraperitoneal positioning of mesh and fixation devices. Additionally, their study showed that the postoperative pain levels, material costs, and hospital stay of the Ventral-TAPP+ cohort were significantly lower than those of the laparoscopic IPOM+ cohort [14].

There were no significant differences in the age distribution between the two groups (p = 0.376), indicating a balanced demographic spread. In the IPOM+ group, 6.1% were under 30 years, 24.2% were between 31 and 40 years, 39.4% were between 41 and 50 years, 18.2% were between 51 and 60 years, and 12.1% were over 61 years of age. In the TAPP+ group, 3.7% were under 30 years old, 40.7% were between 31 and 40 years, 22.2% were between 41 and 50 years, 11.1% were between 51 and 60 years, and 22.2% were over 61 years old. This shows that both techniques were applied across a wide range of age groups without significant bias.

A significant difference in sex distribution was observed, with a higher percentage of females in the IPOM+ group (75.8%) than in the TAPP+ group (44.4%), and a higher percentage of males in the TAPP+ group (55.6%) than in the IPOM+ group (24.2%) (p = 0.013). This gender imbalance could potentially influence outcomes and warrants further investigation. In contrast to our study findings, Megas et al. did not report any significant association between age and sex distribution in either procedure. In addition, the study reported findings comparing laparoscopic IPOM+ and Ventral-TAPP+ procedures. The age of patients in the laparoscopic IPOM+ group (n = 30) was 55.83 ± 11.6 years, while in the Ventral-TAPP+ group (n = 34) it was 54.94 ± 14.70 years, with a p-value of 0.791 [14].

The mean defect size was slightly larger in the IPOM+ group at 2.35 cm (SD = 0.92) compared to the TAPP+ group at 2.06 cm (SD = 0.73), but this difference was not statistically significant (p = 0.18). This indicates that both techniques were used for hernias of comparable size. In unmatched comparisons, the mean hernia size for the laparoscopic IPOM+ group (n = 30) was 3.45 cm^2^ (SD = 1.18), whereas the Ventral- TAPP+ group (n = 34) had a mean hernia size of 2.747 cm^2^ (SD = 0.98), with a p-value of 0.012. Propensity-matched comparisons showed a mean hernia size of 3.35 cm^2^ (SD = 1.17) for the laparoscopic IPOM+ group (n = 27) and 2.98 cm^2^ (SD = 0.945) for the Ventral-TAPP+ group (n = 27), with a p-value of 0.206. Few guidelines have proposed the use of the laparoscopic IPOM+ technique for defect sizes up to 10 cm [15, 16].

The diagnosis type did not differ significantly between the two groups (p = 0.297). In the IPOM+ group, 18.2% had paraumbilical hernias and 81.8% had umbilical hernias. In the TAPP+ group, 29.6% had paraumbilical hernias and 70.4% had umbilical hernias. This similarity suggests that the type of hernia did not influence the choice of the surgical technique. Megas et al. reported similar findings, where epigastric hernias were present in two patients (6.7%) in the laparoscopic IPOM+ group and one patient (2.9%) in the Ventral-TAPP+ group. In addition, combined epigastric and umbilical hernias, there were 2 patients (6.7%) in the laparoscopic IPOM+ group and 4 patients (11.8%) in the Ventral-TAPP+ group. Umbilical hernias were reported in 14 patients (46.7%) in the laparoscopic IPOM+ group and 21 patients (61.8%) in the Ventral-TAPP+ group. In the other cohort, 13 patients (48.1%) in the laparoscopic IPOM+ group and 18 patients (66.7%) in the Ventral-TAPP+ group had umbilical hernias [14].

The duration of surgery was significantly longer in the TAPP+ group than in the IPOM+ group, with a mean duration of 79.26 min (SD = 8.55) for TAPP+ and 70.21 min (SD = 11.90) for IPOM+ (p = 0.002). The longer surgery duration in the TAPP+ group could be attributed to the complexity of the procedure, which requires meticulous dissection and mesh placement in the preperitoneal space. A contrasting finding was reported by Megas et al., with a shorter operating time for the TAPP+ procedure than for IPOM+. Regarding operating time, unmatched comparisons indicated a mean duration of 65.33 min (SD = 25.39) for the laparoscopic IPOM+ group and 57.61 min (SD = 18.36) for the Ventral-TAPP+ group, with a p-value of 0.169. In propensity-matched comparisons, the mean operating time was 65.19 min (SD = 26.43) for the laparoscopic IPOM+ group and 58.65 min (SD = 18.43) for the Ventral-TAPP+ group, with a p-value of 0.303 [14]. However, similar to our study, Sarli et al. reported that TAPP+ took a significantly longer duration to complete the procedure than IPOM+ [12].

Patients in the IPOM+ group reported higher pain scores on postoperative day 0 than those in the TAPP+ group did. The mean pain scores were 4.21 (SD = 0.86) for the IPOM+ and 3.48 (SD = 0.89) for the TAPP+ (p = 0.002). On postoperative day 1, pain scores remained significantly higher in the IPOM+ group, with a mean of 1.91 (SD = 0.58) compared to 1.52 (SD = 0.58) in the TAPP+ group (p = 0.012). These findings suggest that patients undergoing TAPP+ experienced less immediate postoperative discomfort, which could be attributed to the less invasive nature of the preperitoneal approach. Megas et al. reported similar findings regarding postoperative pain assessment. Specifically, they analysed the mean pain scores on the first postoperative day (POD0) at rest and during movement using a 0–10 scale system. In the laparoscopic IPOM+ group, VAS scores were 2.28 ± 1.275 at rest and 3.32 ± 1.49 on movement [14].

The ventral-TAPP+ group exhibited lower pain scores with VAS scores of 1.33 ± 1.18 at rest and 2.26 ± 1.75 on movement. Statistical analysis revealed significant differences in pain levels between the two groups (p = 0.008 at rest and p = 0.023 during movement). Furthermore, the study investigated the maximum pain sensation during hospital stay and found significant differences between laparoscopic IPOM+ and ventral-TAPP+ patients. The maximum VAS score was notably higher in the laparoscopic IPOM+ group (3.76 ± 1.45) than in the ventral-TAPP+ group (2.48 ± 1.58), with a p-value of 0.004, indicating a statistically significant disparity in pain experienced by patients undergoing the procedures [14].

Ruíz et al. suggested that ventral TAPP+ may emerge as the preferred approach for incisional hernia repair. Their study, which involved 59 patients, demonstrated minimal complications. Of the seven patients experiencing complications, one case involved recurrence, another presented with chronic pain, and five cases were classified as complications according to the Clavien-Dindo classification. Additionally, they highlighted extraperitoneal hernia repair as a cost- effective technique, a finding corroborated by the results of our study [15]. Seroma formation was not significantly different between the groups (p = 0.193). In the IPOM+ group, 6.1% of the patients developed seroma, whereas none of the patients in the TAPP+ group developed seroma. This indicates that while seroma formation is a concern, it does not differ significantly between the two techniques. Bittner et al. reported that or eventration of the mesh, seromas, recurrences, and non-restoration of abdominal muscle function [9, 10].

The cost of mesh was significantly higher in the IPOM+ group with a mean of 27,878.79 INR (SD = 2011.80) than in the TAPP+ group, with a mean of 7,444.44 INR (SD = 4,660.25) (p < 0.0001). Similarly, the cost of tackers was significantly higher in the IPOM+ group with a mean of 22,121.21 INR (SD = 2,858.73) compared to the TAPP+ group with a mean of 15,444.44 INR (SD = 9,254.24) (p < 0.0001). This significant cost difference is a crucial factor in clinical decision making, especially in resource-limited settings. Megas et al., in their study, reported significantly lower material costs associated with the preperitoneal method (p = 0.001). Additionally, patients in the ventral- TAPP+ group had a notably shorter length of stay, which was attributed to reduced postoperative pain, decreased reliance on pain medication, and, consequently, faster patient mobilisation. This outcome translated to an indirect cost reduction, encompassing savings on both material and personnel expenditures [14].

Kumar et al. [17] presented findings aligning with the advantages of preperitoneal mesh placement. Although the hernia sizes in their study were similar to ours, the operation times for the e-TEP method were nearly twice as long as those for ventral-TAPP+ in our study (107.52 ± 23.44 min versus 57.61 ± 18.36 min). Ventral-TAPP+ provides surgeons with a clearer view of the surgical site, thus facilitating quicker tissue preparation and defect closure. Furthermore, Kumar’s study using the e− TEP method for small-to-medium-sized ventral hernias reported two recurrences out of 46 cases, highlighting the advantage of TAPP+ as proposed in our study.

At the 3-month follow-up, no cases of seroma or recurrence were observed in either group, indicating good short-term outcomes for either surgical technique. The ventral-TAPP+ cohort, the most contemporary group, was evident in the follow-up duration, averaging 14.70 months, which was relatively shorter than that of the laparoscopic IPOM+ group. However, this timeframe still allows for 1 year of observation, providing insightful results for a promising technique. Throughout this follow-up period, our findings, along with the existing literature, revealed the absence of hernia recurrence [13, 18, 19].

### Implications

These findings suggest that while TAPP+ may involve a longer operative time, it offers the benefits of reduced postoperative pain and lower costs for mesh and tackers. These factors could make TAPP+ a more favourable option in certain clinical scenarios. However, the significant sex imbalance between the groups and its potential impact on outcomes should be considered in future studies. The lower cost associated with TAPP+ also supports its use, particularly in healthcare settings, where cost-effectiveness is a priority.

### Limitations

The significant difference in sex distribution between the two groups could have influenced the results, particularly regarding pain perception and recovery. The study was conducted at a single centre, which may limit the generalisability of the findings. Additionally, the follow-up period was limited to 3 months, which may not capture long-term outcomes, such as recurrence rates and chronic pain. Further multicentre studies with longer follow-up periods are required to validate these findings.

## Conclusion

In conclusion, a comparative study between TAPP+ and IPOM+ for umbilical and paraumbilical hernias demonstrated that TAPP+ is associated with lower postoperative pain and reduced costs for mesh and tackers despite a longer operative time. Both techniques showed no significant differences in seroma formation and had good short-term outcomes, with no recurrences at 3 months. The choice of technique should consider patient-specific factors, including the potential benefits of reduced pain and the costs associated with TAPP+. Further research is needed to explore the long-term outcomes and account for the impact of demographic differences on surgical results.

## Data Availability

The raw data supporting the conclusions of this article will be made available by the authors, without undue reservation.
